# The impact of social networks on knowledge transfer in long-term care facilities: Protocol for a study

**DOI:** 10.1186/1748-5908-5-49

**Published:** 2010-06-23

**Authors:** Anne E Sales, Carole A Estabrooks, Thomas W Valente

**Affiliations:** 1 Faculty of Nursing, University of Alberta, Edmonton, Alberta, Canada; 2 Department of Preventive Medicine, Keck School of Medicine, University of Southern California, Los Angeles, California, USA

## Abstract

**Background:**

Social networks are theorized as significant influences in the innovation adoption and behavior change processes. Our understanding of how social networks operate within healthcare settings is limited. As a result, our ability to design optimal interventions that employ social networks as a method of fostering planned behavior change is also limited. Through this proposed project, we expect to contribute new knowledge about factors influencing uptake of knowledge translation interventions.

**Objectives:**

Our specific aims include: To collect social network data among staff in two long-term care (LTC) facilities; to characterize social networks in these units; and to describe how social networks influence uptake and use of feedback reports.

**Methods and design:**

In this prospective study, we will collect data on social networks in nursing units in two LTC facilities, and use social network analysis techniques to characterize and describe the networks. These data will be combined with data from a funded project to explore the impact of social networks on uptake and use of feedback reports. In this parent study, feedback reports using standardized resident assessment data are distributed on a monthly basis. Surveys are administered to assess report uptake. In the proposed project, we will collect data on social networks, analyzing the data using graphical and quantitative techniques. We will combine the social network data with survey data to assess the influence of social networks on uptake of feedback reports.

**Discussion:**

This study will contribute to understanding mechanisms for knowledge sharing among staff on units to permit more efficient and effective intervention design. A growing number of studies in the social network literature suggest that social networks can be studied not only as influences on knowledge translation, but also as possible mechanisms for fostering knowledge translation. This study will contribute to building theory to design such interventions.

## Background

Despite considerable expenditure on health services in Canada, as in most developed countries, a majority of patients still do not receive care that conforms to current evidence standards [1-9]. This leads to unnecessary illness, suffering, and death, all of which are costly to society. To date, few interventions to implement evidence-based clinical practices have been demonstrated to work consistently across settings, with different provider groups, and different clinical areas [10-12], but have not been well studied in health settings. Social networks are theorized as significant influences in innovation adoption [13-26].

There are several possible paths by which social networks could influence the uptake of knowledge translation interventions. Social networks may affect communication patterns [15,27-31], and are likely to affect the adoption and uptake of information presented in feedback reports. Some psychological factors that may have an impact on how recipients respond to feedback, including perceived behavioral control, may also be associated with position in a social network, and in how accurately people perceive their social networks and the behavior of others in their social networks. The goal of this project is to explore the effects of social networks in long-term care (LTC) nursing units on uptake of a specific intervention--audit with feedback--to improve quality of care in residential LTC settings. We articulate a conceptual model of how social networks may influence intervention uptake, and develop methods to measure their effects.

LTC is relatively understudied, despite expectations that the proportion of Canadians requiring LTC services will grow considerably over the next two decades [32], as is the case in many other countries. LTC settings offer some features that make them attractive places in which to conduct implementation research interventions, particularly audit with feedback interventions. One of the key points from the recent Cochrane review of audit with feedback interventions [33,34] was that while we have insufficient knowledge about how best to design effective audit with feedback interventions, settings with relatively little prior exposure to these interventions, such as in LTC, may be more receptive to them. Similarly, over time, repeated unchanged audit with feedback may cease to be effective even if it was effective initially. In LTC settings, the existence of readily available audit data, described below, makes it feasible to conduct this type of intervention at relatively low cost.

### Some types of data are more available in LTC than other sectors

The *Resident Assessment Instrument-Minimum Data Set version 2.0 (RAI-MDS 2.0) *is an international system to collect essential information about the health, physical, mental, and functional status of nursing home residents [35-43]. It consists of several assessment modules, including an initial or admission assessment, annual assessment, quarterly assessments, and assessments for major health-related events. The full assessment, used on admission and annually, includes sections on demographics, health problems, and functional status. A less extensive assessment is conducted quarterly to evaluate change in status. RAI-MDS 2.0 is widely used throughout many countries, and is currently being implemented in many Canadian jurisdictions.

### Audit with feedback interventions are an efficient way to use existing data

Audit with feedback consists of two components: the audit of data containing indicators of outcomes or processes of care, ideally linked to quality of care; and delivery of reports or communications that present these data to care providers in a format that can be understood and used for quality improvement. Audit with feedback interventions have been widely used in healthcare settings to promote use of evidence based practice or implement guidelines [33,34].

The theory guiding feedback interventions is based on concepts of intrinsic and extrinsic motivation as well as social influence. In work settings that rely on teams to conduct work, social comparison may play a role in team performance, where members of the team make comparisons both inside and outside of the team. These conditions apply quite generally to healthcare environments, where care is typically delivered through teams, teams are usually hierarchical rather than egalitarian, and there is constant performance comparison across teams.

### Social networks may be one reason for inconsistent effect of feedback interventions

Social networks are theorized as significant influences in innovation adoption and behavior change [13-26]. Seminal research has explored the role of social networks in disseminating knowledge among a wide variety of groups, including farmers, women in developing countries, public health officers, and physicians [15,23,44]. A class of interventions designed to promote knowledge translation, using opinion leaders, uses aspects of social network theory to foster planned behavior change or knowledge translation [17,20,25-27,45-56]. Despite a history of interest in social network theory, and empirical work exploring the influence of social networks, as well as attempts to use social networks in interventions, our understanding of how social networks operate within healthcare settings is limited because of the paucity of studies in the area. As a result, our ability to design optimal interventions using social networks to foster knowledge translation may also be limited.

At its core, social network theory is quite intuitive. It postulates that humans are social in nature, and one consequence of their social being is to exist in relationship to each other. One way of characterizing the relations among humans is to characterize networks of interactions that bind humans together in social structures [31,57-60]. However, in healthcare settings, different types of providers tend to have very discipline-specific networks. Even in the highly structured environment of hospital work, it appears that people do not know each other across disciplinary boundaries [28,61-67].

In a study of social networks in a LTC setting, Cott [66,67] assessed social networks among different disciplines and across three units in a large Toronto LTC organization. Cott elicited responses from participants using name recognition (providing lists of all staff members on each unit), asking eight questions: whether they knew the other team member; whether they chatted casually with the other person; received information from the other person; gave information; problem-solved together; planned to work together; helped each other with work; and had lunch or coffee together. Cott concluded that patterns of decision making within all three units was very hierarchical, with higher status professionals making decisions, and lower status staff responsible for carrying these decisions out in terms of daily care. The implications of this study for teamwork among and across disciplines are quite striking, and the detail provided through the careful delineation of network structures suggests that this is a fruitful approach to acquiring important information about the flow of information.

**Figure 1:**
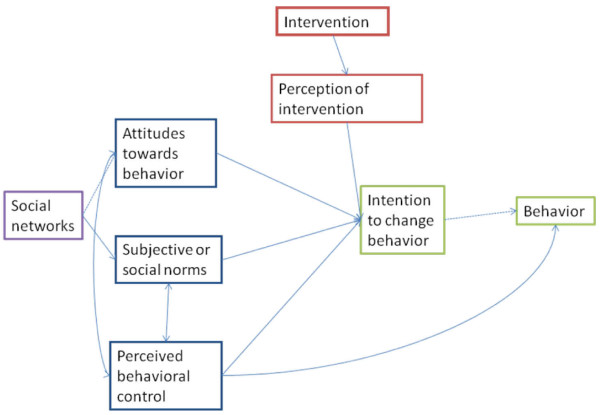
**Possible paths by which social networks might affect uptake of feedback report**. Note that the three boxes on the left (attitudes towards behavior, subjective or social norms, and perceived behavioral control) as well as intention to change behavior and behavior are all primary components of the Theory of Planned Behavior. We have added the social networks box to the left of the three predictors of intention to change behavior, as well as the intervention and perception of intervention boxes to show where we believe social networks are likely to exert effect.

In Figure 1, we describe possible paths by which social networks could influence uptake of feedback reports. Social networks affect communication patterns [15,27-31], and are likely to affect the diffusion and uptake of information presented in feedback reports. Some psychological factors that appear to have an impact on how recipients respond to feedback, including perceived behavioral control, also appear to be associated with social network position, and attitudes and dispositions of social network members. We propose that social networks influence key elements in the Theory of Planned Behavior (TPB) [68-70], which is being used in several implementation studies (blue-bordered boxes in Figure 1). It provides a reasonable basis for understanding how individuals form an intention to change behavior, which has been demonstrated to have a relatively strong association with actual behavior change [71]. In this framework, we suggest that social networks may influence social norms, which are a key construct in the TPB, as well as exerting a direct influence on whether or not staff members perceive the feedback reports to be useful. Network influence can be positive, enhancing the likelihood of the staff member using the feedback report, or negative, making it less likely that the feedback report will be used. When influential others within social networks have negative opinions of an innovation, it is less likely to be adopted, or at least, forming an intention to use a feedback report if influential others recommend against using it can create conflict in the individual. By including questions on our post-feedback survey that measure what respondents believe others think, and how they perceive this to affect them, we will be able to assess the degree of conflict posed by negative social network influences. We believe that combining data collection based on the TPB with social network data collection will allow us to address key questions about the effects of social networks on uptake of feedback reports.

## Methods and design

Our specific aims include:

1. To collect social network data among staff in two LTC facilities.

2. To use quantitative social network analysis to characterize social networks in these units.

3. To describe how social networks influence uptake and use of feedback reports based on RAI-MDS 2.0 data.

### DICE: The parent intervention study

In the Data for Clinical Improvement and Excellence project (DICE), we are delivering feedback reports tailored to all direct-care staff (care managers, RNs, LPNs, nurse aides or personal care attendants, rehabilitation specialists such as occupational or physical therapists, pharmacists, and social workers, as well as senior managers and administrators) in four nursing homes in Edmonton, Alberta. The purposes of this study are to assess feasibility and methods of constructing feedback reports on a monthly cycle, deliver these reports to staff, and assess staff response to the reports. Feedback reports document processes of care linked to modifiable outcomes. Examples of processes of care measured through RAI-MDS 2.0 include plans related to promoting continence, nutritional problems, oral care, or skin treatments. Related outcomes include unmanaged pain, continence status, or presence of pressure ulcers. The specific items included on the feedback reports are assessment of pain among residents on a unit; depression screening; falls risk; and actual falls within the previous 31 to 180 days. Delivery of feedback reports began in January 2009, with monthly reporting for 13 months.

### Settings

Two of the four nursing homes included in the parent intervention project are part of a large, publicly funded, LTC organization in Edmonton, Alberta. The two facilities included in this study have been collecting RAI-MDS 2.0 data longer than the other nine facilities in this organization. The first of the two LTC facilities is larger than the other, with four care units and 149 continuing care beds, while the smaller of the two has two care units and 75 continuing care beds. Each facility also provides specialized services; the units providing these specialty services will not be included in this study. The RAI-MDS 2.0 assessments providing the data used in the feedback reports are conducted in continuing care units only. Both facilities provide a full range of LTC and rehabilitation services through interdisciplinary teams.

These two facilities, despite being part of the same organization, have distinct characteristics that will enhance applicability of the findings of this study to other LTC settings. The larger of the two is an older facility. The physical layout of units is similar to traditional hospital nursing unit structure, with long hallways off a central corridor. Resident rooms are mostly semi-private. Nursing stations are located midway down each hallway, with large central gathering spaces for residents in the central hub area on each of the two floors. Staff space is limited, and staff spend most of their time out in resident rooms or in the central areas with residents. The smaller facility is a much newer facility. The care units are organized in a circular plan, with access to both central areas and smaller spaces for more privacy. While there are no traditional nursing stations, there is space for staff to engage in care planning and organization while maintaining visual contact with resident areas. Staff in the two facilities differ in age and other characteristics, with older staff on average at the larger site, and younger staff at the smaller.

### Sample

All employed staff providing direct care to residents in continuing care in both facilities are eligible to participate in the study. Direct care staff include care managers (unit managers), RNs, LPNs, nurse aides or healthcare aides, rehabilitation specialists including occupational, recreational, and physical therapists and their assistants, pharmacists, dietitians, and social workers. Based on the pilot project currently underway, we anticipate recruiting at least 60% of healthcare aides, 60% of LPNs, 60% of RNs, and 75 to 100% of rehabilitation staff, pharmacists, social workers, and dietitians to participate in the interviews following the audit with feedback intervention. We anticipate that most of these participants will also agree to participate in the social network surveys, and we are including compensation to facilitate participation, both for the facility and for respondents. In our pilot study, during the first four hours of data collection, we recruited 53 out of a possible 200 staff participants who completed a 15- to 20-minute paper and pen survey. Staff were enthusiastic and eager to participate, and several nurse aides said that this was the first time researchers had ever included them in a research study.

We will also conduct interviews with senior managers at each site to obtain their assessment of the networks among staff in the facility, and the impact of those networks on adoption of innovation and change, based on prior efforts to introduce new practices. There are a total of six senior managers between the two sites.

### Sample size

Based on our pilot project, we will have a sample of approximately 50 to 60 staff participating in DICE at each facility. This represents about 80% of all direct-care staff providing care on day or evening shift. We expect that at least 70% of these staff will participate in the social network surveys, which would yield about 40 staff, or slightly over 50% of all staff on those two shifts, responding to these surveys. Missing data are a persistent problem in social network analysis as in other social science and health services applications. Use of lists or name recognition questionnaires will help to decrease this problem of missing data. There is some literature that suggests that 50% response rates are sufficient for robust specification of social networks, and we will also evaluate whether we can use assumptions based on mutuality or reciprocity of ties to impute missing data [72-76]. We will offer multiple days and times for responding to the social network questionnaires, offer refreshments to all respondents as they complete the questionnaires, and work with facility leadership to backfill staffing to allow staff members to participate. We will be offering backfilling as part of the larger DICE study, and we will provide extra backfilling staff during the two periods when we plan to collect network data. We expect to have a total of at least 85 to 100 staff participating in the network surveys, out of over 200 total staff respondents we expect in the full DICE project.

Low response rates have created problems in prior work on social networks in health services research. We believe that the fact that the direct care staff will know our research staff well by the time we ask them to complete the social network surveys will enhance response rates. Research assistants will be known and trusted by the time we approach them to participate in this added component. In addition, this will be a part of an ongoing intervention--the audit with feedback reports--in which staff will have an ongoing relationship with our research team. We have had outstanding response to our initial work in these two facilities, and continue to engage in a mutually respectful relationship.

### Procedures in DICE

We obtain RAI-MDS 2.0 data from the organization's corporate office on a monthly basis. Assessments are conducted quarterly for each resident, but resident assessments are staggered so that roughly one-twelfth of all residents are being assessed each month. We distributed monthly feedback reports to staff individually in the facilities, beginning in January 2009. We followed most rounds of feedback reports with surveys of members of all provider groups to ask about actions taken to modify care processes, with specific emphasis on the aspects of care included in the feedback reports. A sample post-feedback survey is attached in Additional file 1. A key component of this survey is the inclusion of items designed using the TPB [68-70]. There is considerable evidence about how well intentions predict observed behavior [71]. In addition to using the survey items designed using TPB, we are also asking respondents to discuss ways they would plan to use information in the feedback reports. In addition to self-report surveys, we will be conducting observations using time sampling to assess occurrences of discussion of feedback reports and observed changes in practice, following feedback report distribution. We will conduct trend analyses of the monthly data, and provide trend data, not just cross-sectional data, in later iterations of the feedback reports.

As part of the larger DICE study, we have also collected data on participant perceptions of organizational context, using the Translating Research in Elder Care (TREC) survey [77,78] which is a suite of survey instruments that, in addition to assessing organization context with the Alberta Context Tool (ACT [79]), measures a variety of ways in which facilitation processes may be used to improve quality of care. Of importance to this study, the TREC survey also asks respondents to describe their assessment of job satisfaction, burnout, and research utilization. These items will be used to triangulate across other data (such as the post-feedback survey and observational data) to assess validity and reliability of findings. The ACT itself is designed to measure leadership, culture, approaches to evaluation (how staff perceive the organization using data to assess its performance), structural resources, human resources, social capital, and time and space resources for getting work accomplished, as well as for quality improvement and knowledge uptake, and is embedded within the TREC survey. The survey will be administered using a facilitated web-entry process, or paper-based administration, depending on what is feasible for the participant. We will ask all direct-care staff, as well as managers, to complete the survey once at baseline and again at the end of the intervention period.

### Procedures for social network data collection

This is a prospective study, with primary data collection on work-related social networks, using social network analysis techniques to analyze the data and characterize social networks. These data will be combined with individual level data from DICE to explore the impact of social networks on individual uptake and use of feedback reports. We will analyze the data on social networks using both graphical and quantitative techniques to characterize attributes of the networks. We will include two methods of capturing social network data in the units included in the study.

### Questionnaires to elicit social networks related to feedback reports

We will obtain lists of all staff working on each unit from managers at each facility. Using these lists, which will be current for the weeks before and after feedback report distribution, we will ask staff to check off the names of all staff members with whom they have: worked in the last two weeks; have talked at least once a day; discussed resident care with; gone to for advice about work issues; and discussed the feedback reports. In addition, each participant will be asked to rate whether any discussion of the feedback report was generally positive, neutral, or generally not positive, for each staff member with whom they discussed the report. We will also allow space for respondents to include a staff member or someone who does not work in the facility as a member of their network. A draft questionnaire is included in Additional file 2.

Questionnaires of this type, often called roster and/or recognition questionnaires, provide more complete data than those that use free recall (such as the Hiss instrument), in studies where completeness of network data has been assessed using more than one type of questionnaire [80,81]. Participants find the task of reporting ties for each question easier, and a larger number of ties are reported than with free recall questionnaires. Validity and reliability of this type of questionnaire response have been evaluated in prior studies, and although those results cannot be automatically generalized to this study, findings are usually that reliability is high, as is validity, using multitrait-multimethod approaches [80-84].

The questions included in the questionnaire are adapted from questions used in previous studies of social networks. They will be piloted prior to administering the surveys among staff in a LTC facility not included in this study to ensure that the language is understandable to all staff and to assess how long it takes to complete the survey. Each list will include spaces to add names of staff members from other units if appropriate, although we will not include names of staff outside each unit in the prepared lists. A few network questions provide a wealth of data that can be used to determine the centrality of participants, strength of their ties, and degree of mutuality.

All social network questionnaires will be administered using paper and pen, and participants will be given privacy to complete the questionnaire, either at work or at home, and an envelope to return the completed form by mail. Completion of the questionnaire will be voluntary, and respondents will be assured that no one in the facility will have access to their answers. It will not be possible to administer these questionnaires anonymously, because we will need to use the names of respondents and staff members, but we will assure that all names are coded as soon as we enter the data into the database, and original questionnaires will be stored in a locked space at the university.

We will also ask demographic questions, including gender, English as a first language, ethnic background, age, formal schooling, and how long they have been working both in LTC and on this particular unit.

### Analysis

Our research questions are:

1. Are the characteristics of individuals' networks associated with the likelihood that they will report intent to use the information in the feedback reports to change their behavior in caring for residents?

2. Do social networks with more positive interactions about the feedback reports increase the likelihood that individuals will report intent to use the information in the feedback reports to change behavior?

We will use the post-feedback surveys data to assess uptake of the feedback reports, as well as factors facilitating or inhibiting their uptake. We will analyze the trends in the feedback reports as outcomes, with the reports themselves as the primary outcome in each subsequent month, similar to the approach we used in a previous study to assess factors affecting intervention processes [85]. While this is primarily a descriptive approach, it provides temporal linkage between intervention events and trended outcomes. In addition, we will use thematic coding of comments and responses to less structured questions to assess emergent themes described by staff as affecting their use of the feedback reports.

The primary analysis will use the question asking respondents if they intend to change behavior based on the feedback report. This ties into the theoretical framework we present in Figure 1, which combines the TPB [68-70] with influences from social networks. We will analyze these data at the level of individual staff member in each of the six units included in the study. The key independent variables in the equations predicting intent to change behavior will be two variables derived from the network data: in-degree centrality and the valence of the network members' attitudes toward the feedback reports. Other variables in the model include the other constructs of the TPB, measured in the post-feedback survey, respondent age, type of provider, and years of experience on the nursing unit. We will dichotomize the dependent variable, and estimate it using multivariable logistic regression using multi-level modeling techniques.

To assess how networks affect uptake of feedback reports, we will estimate individual-level models adjusting for unit level through the cluster command in Stata version 10, including the nursing unit on which the staff member works. We are constrained by the small number of two facilities and six units, which prevents us from using full multi-level modeling techniques that require larger numbers of observations at the higher levels (unit and facility). However, the cluster command will correct standard errors and ensure efficient and unbiased coefficient estimates. We will estimate models for two primary outcomes: intent to use the feedback report, as measured by the TPB questions on the survey; and the single item on the post-feedback survey asking whether the respondent has used the feedback report. The TPB items will be scored using the approach outlined by Francis *et al. *[70].

To address the first research question, we will aggregate characteristics of the social networks of individuals in the study and attribute them to each individual. We will focus on responses to the following question on the network survey (Additional file 2): Who do you go to for advice about work issues? We will estimate the in-degree centrality for each individual in each of the five networks. In-degree centrality measures the number of ties that are directed to a single individual, and can be calculated for each individual in a network by adding together the number of times a person is mentioned by others. We will use this measure of network centrality because it validly measures an opinion leadership role and is one of the most stable measures of centrality, even when only 50% of respondents complete the survey [86]. We will include this variable, attributed to the individual level, as a regressor in two equations, one estimating the single item response to intention to use the information in the report, the other using the more complex variable including other items on the TPB survey.

To address the second research question, we will use question five on the network survey: 'Who have you discussed the feedback report with?' This is followed by a three-point scale for each person on the list of unit staff: 'Discussion made me feel: positive, neither positive nor negative, negative.' We will score this scale +2 for 'positive', +1 for 'neither', and -1 for 'negative.' We will use these data to calculate the valence of the network exposure to the feedback reports. This provides an individual-level measure of each person's social environment derived from their social networks.

Our secondary analyses will focus on network analytic techniques. As Luke and Harris describe in their overview of applications and methods for network analysis in public health [87], the three primary approaches to analyzing network data are: visualization using graphic display; network description, describing and characterizing networks among staff in these units [67]; and use of both blockmodeling [88-93] and stochastic methods to build and test hypotheses [94,95]. Our analyses will apply the first two approaches with some preliminary use of stochastic modeling techniques, primarily to assess feasibility for using these techniques in future research. It is unlikely that the sample size in this study--two nursing homes, six units, and up to 210 staff members--will be sufficient for robust modeling using multivariate stochastic techniques.

We will use the network configuration that results from responses to question five on the questionnaire: 'Who have you discussed the feedback report with?' This aspect is most directly related to our primary research objective, understanding how social networks affect uptake of the feedback report. We will use a program called UCINET [96] for the analysis of network data. UCINET has the capacity to graph network data for visualization, and to perform blockmodeling as well as analysis using p* estimators, which have been developed for network analysis. We will estimate several measures of network centrality [86,97-101], as well as explore the relative density of the network [102-105]. We will assess the presence of weak ties, or bridges, between different sub-networks [57,106-111].

We will attempt to explore the relationship between measures derived from network analysis and attributes measured at the individual, nursing unit, or facility level [112-121]. An issue that will require careful consideration is how to characterize networks within the multi-level context of nursing units and organizational structures. In other words, the network configurations evident in the data may be a product of organizational factors that we cannot disentangle given the small number of organizational units. We will attempt to use multitrait-multimethod approaches to assess the validity and reliability of the data [80,83] by combining data from the survey responses with observational data.

## Discussion

### Expected outcomes and links to future research

#### Opportunities for knowledge translation theory-building using social network data

A growing number of studies in the social network literature suggest that social networks can be studied not only as determinants of knowledge translation or information dissemination, but also as mechanisms for inducing information dissemination [102,105,122-126]. Opinion leader interventions are one such approach, albeit a relatively weak one that relies on existing opinion leaders and their current positions within their networks. A more proactive approach might involve coaching opinion leaders to extend their influence by actively encouraging them to fill holes in their networks, for example, or strengthening key bridges or ties. This kind of intervention would involve feeding back data on network structure and function to network participants, could include coaching or education in methods of network creation, and then measuring differences before and after the intervention in information dissemination patterns. We do not propose to include this kind of feedback in our present project, but it may be feasible in the future.

We also expect this research to be useful in our understanding of social networks and how they influence a wide variety of activities within work settings. Healthcare is an increasingly important sector of the economy, with specific characteristics such as hierarchical structure, the existence of multiple, often competing professional groups, and contested evidence (to name just a few). Understanding the functioning of social networks in these settings also may provide knowledge that generalizes outside healthcare.

#### Knowledge translation and exchange

The principal audience for this work is the community of knowledge translation researchers. Drs. Sales and Estabrooks are active members of several different communities among knowledge translation researchers in Canada and internationally, and Dr. Valente is active in several different groups of researchers focused both on social network theory and analysis, and implementation science, in the United States and internationally. Both Drs. Sales and Estabrooks, notably, are lead researchers in the newly funded KT Canada project (CIHR; PIs Grimshaw and Straus), which will offer at least annual venues for disseminating the findings of this study before publication in peer-reviewed journals. In addition, both have been active participants in the Knowledge Utilization Colloquium, an international group of knowledge translation scholars who represent groups in Canada, the United Kingdom, Sweden, Australia, and the United States. This group meets annually also, and we will have an opportunity to disseminate findings through the venues they offer.

Beyond KT researchers, we believe our findings will be of interest to social scientists more generally. As we noted above, we think it likely that insights we gain into the functions of social networks in LTC settings are likely to be of use in other settings and sectors.

In terms of the Knowledge to Action cycle [127], depicted on CIHR's web site http://www.cihr-irsc.gc.ca/e/33747.html, we believe this research currently is part of knowledge inquiry, at the top of the triangular wedge representing knowledge creation. We expect that our findings will readily spur future work focused on adapting knowledge to local contexts, assessing barriers to knowledge use, and selecting, tailoring, and implementing interventions; but we believe that these activities will take longer and will require future research efforts. Our work currently is highly embedded within the organizations in which we are doing the audit with feedback interventions in the DICE program, and our team for that project is one-half decision-makers and one-half researchers. As a result of the fact that this application is intended to be embedded within the larger project, we believe there will be natural exchange with decision-maker partners.

## Competing interests

The authors declare that they have no competing interests.

## Authors' contributions

AS is the principal investigator for this funded study; CE is co-investigator, and TV is a key collaborator. AS took the lead in drafting the text; CE and TV both critically reviewed it and contributed to the study proposal on which it is largely based. All authors read and approved the final manuscript.

## Supplementary Material

Additional file 1 **Post-feedback survey used in the DICE study**. Click here for file

Additional file 2 **Social network survey used in this study**. Click here for file
