# Supplementary material for: The impact of social networks on knowledge transfer in long-term care facilities: Protocol for a study
Source: Implement Sci. 2010 Jun 23;5:49. doi: 10.1186/1748-5908-5-49 (PMC2900220; doi:10.1186/1748-5908-5-49)
Supplement: Additional file 2 — Social network survey used in this study. [file 1748-5908-5-49-S2.DOC]

**Additional file 2**

**Social Network Survey**

**OBJECTIVE:**

We would like to know who among your co-workers you spend time with or go to for certain kinds of information or help.

**INSTRUCTIONS:**

This survey has two sections. In Section I, we provide a list of the names of people that work on your unit and we ask 6 questions about the way you interact with them. In Section II, we ask you a few questions about yourself.

In Section I:

1. Please **circle** your name first on the list of staff names.

1. Place an **“X”** in the box for each person on the list that you interact with in the specific way we are asking about.

*For example*: for Question 1- place an “X” in the box for everyone you worked during a shift with on this unit during the last two weeks.

1. Please fill in any names of people in the space provided for each question if they are not on the list.

*For example*, if someone usually works on a different unit but worked with you on this unit **during the last two weeks**, add his/her name in the first column.

**SECTION I:**

Please **circle** your name first.

Place an **“X”** in the box for each person who you:

| Staff Name | 1. worked with **the past 2 weeks** … | 2. talk with at least **once per day** … | 3. discuss care of residents with … | 4. go to for advice about work issues… |
| --- | --- | --- | --- | --- |
| 1. Staff1 | □ | □ | □ | □ |
| 2. Staff2 | □ | □ | □ | □ |
| 3. Staff3 | □ | □ | □ | □ |
| 4. Staff4 | □ | □ | □ | □ |
| 5. | □ | □ | □ | □ |
| 6. | □ | □ | □ | □ |
| 7. | □ | □ | □ | □ |
| 8. | □ | □ | □ | □ |
| 9. | □ | □ | □ | □ |
| 10. | □ | □ | □ | □ |
| 11. | □ | □ | □ | □ |
| 12. | □ | □ | □ | □ |
| 13. | □ | □ | □ | □ |
| 14. | □ | □ | □ | □ |
| 15. | □ | □ | □ | □ |
| 16. | □ | □ | □ | □ |
| …… | □ | □ | □ | □ |
| Please add names below if the person is not on the list and put “X” in the corresponding box if you do that activity with this person. | | | | |
|  | □ | □ | □ | □ |
|  | □ | □ | □ | □ |
|  | □ | □ | □ | □ |
|  | □ | □ | □ | □ |

Please go to the next page.

Please **circle** your name first.

Place an **“X”** in the box for each person who you:

| Staff Name | 5. Discussed the feedback report you received last week with… | 6. When you discussed the feedback report, the discussion with each person **Made me feel positive about the report, Made me feel negative about the report** or **Neither positive nor negative** by placing an “X” in one of the boxes below: | | |
| --- | --- | --- | --- | --- |
|  |  | **Made me feel positive about the report** | **Made me feel negative about the report** | **Neither positive or negative** |
| 1. Staff1 | □ | □ | □ | □ |
| 2. Staff2 | □ | □ | □ | □ |
| 3. Staff3 | □ | □ | □ | □ |
| 4. Staff4 | □ | □ | □ | □ |
| 5. | □ | □ | □ | □ |
| 6. | □ | □ | □ | □ |
| 7. | □ | □ | □ | □ |
| 8. | □ | □ | □ | □ |
| 9. | □ | □ | □ | □ |
| 10. | □ | □ | □ | □ |
| 11. | □ | □ | □ | □ |
| 12. | □ | □ | □ | □ |
| 13. | □ | □ | □ | □ |
| 14. | □ | □ | □ | □ |
| 15. | □ | □ | □ | □ |
| 16. | □ | □ | □ | □ |
| …… | □ | □ | □ | □ |
| Please add names below if the person is not on the list and put an “X” in the corresponding box if you do that activity with this person. | | | | |
|  | □ | □ | □ | □ |
|  | □ | □ | □ | □ |
|  | □ | □ | □ | □ |
|  | □ | □ | □ | □ |

Please go to the next page.

**SECTION II:**

We would like some information about you. Please respond to the following questions; if you are uncomfortable answering any question, please feel free to leave it blank.

1. What is your gender?

□ Male

□ Female

1. Is English your first language (that is, is English the language you grew up speaking)?

Please choose one:

□ Yes

□ No

If No, Please specify the language: _______________________________

□ I grew up speaking English and _________________________________

1. What is your ethnic background? Please specify all that apply:

□ Canadian

□ Other, please specify: __________________________________________

__________________________________________

1. Please tell us what age group you are in:

□ Under 25

□ 25-30

□ 31-35

□ 36-40

□ 41-45

□ 46-50

□ 51-55

□ 56-60

□ 61-65

□ Over 65

1. How much formal schooling have you completed?

□ Some high school

□ High school (Canadian equivalent)

□ Some post-secondary school (college or university)

□ College degree (2-3 years)

□ Undergraduate university degree (4 years)

□ Graduate diploma or degree

1. How long have you been working in a long term care setting?

If more than 1 year**,** how many years? ___________ (example: 7 years)

or

If less than 1 year, how many months? ___________ (example: 11 months)

1. How long have you been working on this unit?

If more than 1 year, how many years? __________ (example: 2 years)

or

If less than 1 year, how many months? __________ (example: 5 months)

THANK YOU FOR COMPLETING THE SURVEY!

Please use the space below for comments if you have any.
